# A Biosimilarity Study Between QX001S and Ustekinumab in Healthy Chinese Male Subjects

**DOI:** 10.3389/fphar.2021.675358

**Published:** 2021-05-18

**Authors:** Lei Gao, Qingmei Li, Hong Zhang, Min Wu, Min Fang, Lizhi Yang, Xiaojiao Li, Jingrui Liu, Cuiyun Li, Hong Chen, Xiaoxue Zhu, Yanhua Ding, Mingwei Zhou

**Affiliations:** ^1^ Phase I Clinical Research Center, The First Hospital of Jilin University, Changchun, China; ^2^ The Department of Pediatric Nephrology, The First Hospital of Jilin University, Changchun, China; ^3^ Qyuns Therapeutic Co. Ltd, Taizhou, China; ^4^ Nanguan District Maternal and Child Health and Family Planning Service Center of Changchun, Changchun, China; ^5^ Department of Dermatology, China-Japan Union Hospital of Jilin University, Changchun, China

**Keywords:** ustekinumab, biosimilar, immunogenicity, pharmacokinetics, inter-subject variability

## Abstract

**Objective:** To evaluate the tolerance, variability, and pharmacokinetics (PK) of QX001S, a biosimilar for ustekinumab, in healthy Chinese men.

**Methods:** One hundred and seventy-eight healthy men were recruited in this randomized, double-blind, single-dose, two-arm, parallel study, and received 45 mg of QX001S or ustekinumab in a single subcutaneous injection. PK, immunogenicity, and tolerance were evaluated in all participants for a period of 113°days.

**Results:** The similarity between the two drugs was determined by comparing the baseline characteristics for each drug. The PK parameters were similar in the two groups: QX001S (*n* = 89) and ustekinumab (*n* = 88). The 90% confidence intervals (CIs) for the geometric mean ratio (GMR) of QX001S to the reference (ustekinumab) for the maximum observable serum concentration (C_*max*_), area under the curve (AUC) from zero to the final quantifiable concentration (AUC0–t), and AUC from zero to infinity (AUC_0–∞_) were 100.90–118.68%, 98.71–115.26%, and 98.49–115.81%, respectively, which were within the predefined bioequivalence limit of 80.00–125.00%. High inter-subject variability (ranging from 32.0 to 33.5%) was observed. A total of 17 participants (19.1%) in the QX001S group and 36 (40.9%) in the ustekinumab group developed anti-drug antibodies (ADA) after administration. Nevertheless, the ADA did not affect the outcomes of the bioequivalence tests. Adverse reactions were recorded in 38 individuals injected with QX001S and 37 injected with ustekinumab. The most common adverse reactions were upper respiratory infection and elevated alanine aminotransferase.

**Conclusions:** Our study ratified pharmacokinetic biosimilarity between QX001 S and ustekinumab, with high variability between subjects.

## Introduction

Biological products specifically refer to various large and complex molecules produced by living cells such as microorganisms. Due to their molecular complexity and production processes, biological products are often more difficult to characterize than traditional small molecular drugs (European Medicines Agency (EMA), 2014; US Food and drug Administration (FDA), 2015; World Health Organization (WHO), 2015). Biological products have been successfully used to treat many life-threatening and chronic diseases globally. Due to their high production cost, the clinical application of biological products is usually limited by their expensive price, particularly in developing countries. To overcome this obstacle, a variety of alternative approaches have been developed, including biosimilars, which are biologic medical products that are highly similar to already approved biological medicines. Biosimilars can improve the overall health of patients by improving patient accessibility and reduce medicine-related costs (Chopra and Lopes, 2017).

A step-by-step protocol for developing biosimilars was published by the Food and Drug Administration of European Medicines Agency and US Food and Drug Administration (European Medicines Agency (EMA), 2014; US Food and Drug Administration (FDA), 2015). Briefly, 1) the biological functions of the biosimilar should be evaluated and compared with those of the reference drug. 2) The similarity of the pharmacokinetic (PK) and pharmacodynamic (PD) characteristics of the biosimilar should be evaluated. 3) The similarity between the biosimilar and its reference should be evaluated with the clinical patient population, including the treatment effect, drug safety, and immunogenicity. The dosage and route of administration for the reference drug should also be referenced (European Medicines Agency (EMA), 2014; US Food and Drug Administration (FDA), 2015; World Health Organization (WHO), 2015).

Ustekinumab is a human IgG1қ monoclonal antibody against cytokines including interleukin (IL)-12 and IL-23. Ustekinumab can specifically bind to the p40 protein subunit of IL-12 or IL-23 with high affinity to reduce the activity of these cytokines. Ustekinumab has been used to treat adult patients (18°years or older) with moderate to severe plaque psoriasis and Crohn’s disease (Centocor Ortho Biotech Inc, 2020; Yoshihara, et al., 2021). Approximately 125 million people worldwide have psoriasis, which often causes substantial morbidity and increased rates of inflammatory arthritis, cardiometabolic diseases, and mental health disorders (Armstrong and Read, 2020). Therefore, there is an urgent need for effective therapies with biomedicines such as ustekinumab. In addition, the availability of biosimilars is necessary to make drugs more affordable for patients at relatively low prices.

Biosimilars of ustekinumab have been actively developed globally, including in China. To evaluate the characteristics of QX001S, a biosimilar of ustekinumab, QX001S was prepared using Chinese hamster ovary cells (CHO cells). The amino acid sequence of QX001S was found to be exactly the same as that of ustekinumab. Furthermore, the structure, physicochemical properties, biological activity, and stability of QX001S were also highly similar to those of ustekinumab, except for a difference in charge distribution caused by N-sugar modification. An ongoing study focused on the preclinical pharmacology, toxicology, and PK characteristics of QX001S revealed that QX001S is highly similar to ustekinumab with respect to the PD, PK, and toxicology (data not published), implying the clinical application potential of QX001S.

To evaluate the bioequivalence of a biological analogue and its reference products, clinical PK studies are required (CHINA Food and Drug Administration, 2015). To this end, a single-dose PK study was conducted with healthy Chinese men to assess the bioequivalence of QX001S and ustekinumab. Healthy individuals were recruited for this study rather than patients because confounding factors such as disease-related variability, comorbidity, and concomitant treatment are more likely to be absent in healthy individuals. The therapeutic dose of the reference drug in clinical trials was between 45 and 90 mg (Centocor Ortho Biotech Inc, 2020). Therefore, 45 mg was selected in accordance with the dosage used in earlier clinical trial conducted by the sponsor.

In this study, the PK profiles of QX001S were analyzed and compared with those of ustekinumab. Furthermore, the tolerability, safety, and immunogenicity of QX001S were also evaluated.

## Methods

### Study Design and Participants

This study was carried out in the phase I Clinical Research Center of the First Hospital of Jilin University for 1°year from 2018–11–21 to 2019–12–16 (Chinese Clinical Trial Registry, Registration No. CTR20181,658; http://www.chinadrugtrials.org.cn/index.html). The protocol for this study was approved by the ethics committee of our hospital. Our study was conducted in agreement with the guidelines of the Declaration of Helsinki and the International Conference on Harmonization Good Clinical Practice Guidelines and fulfilled local regulatory requirements. The number of the protocol is QX001SA-01. Written informed consent was obtained from all participants in this study.

**FIGURE 1:**
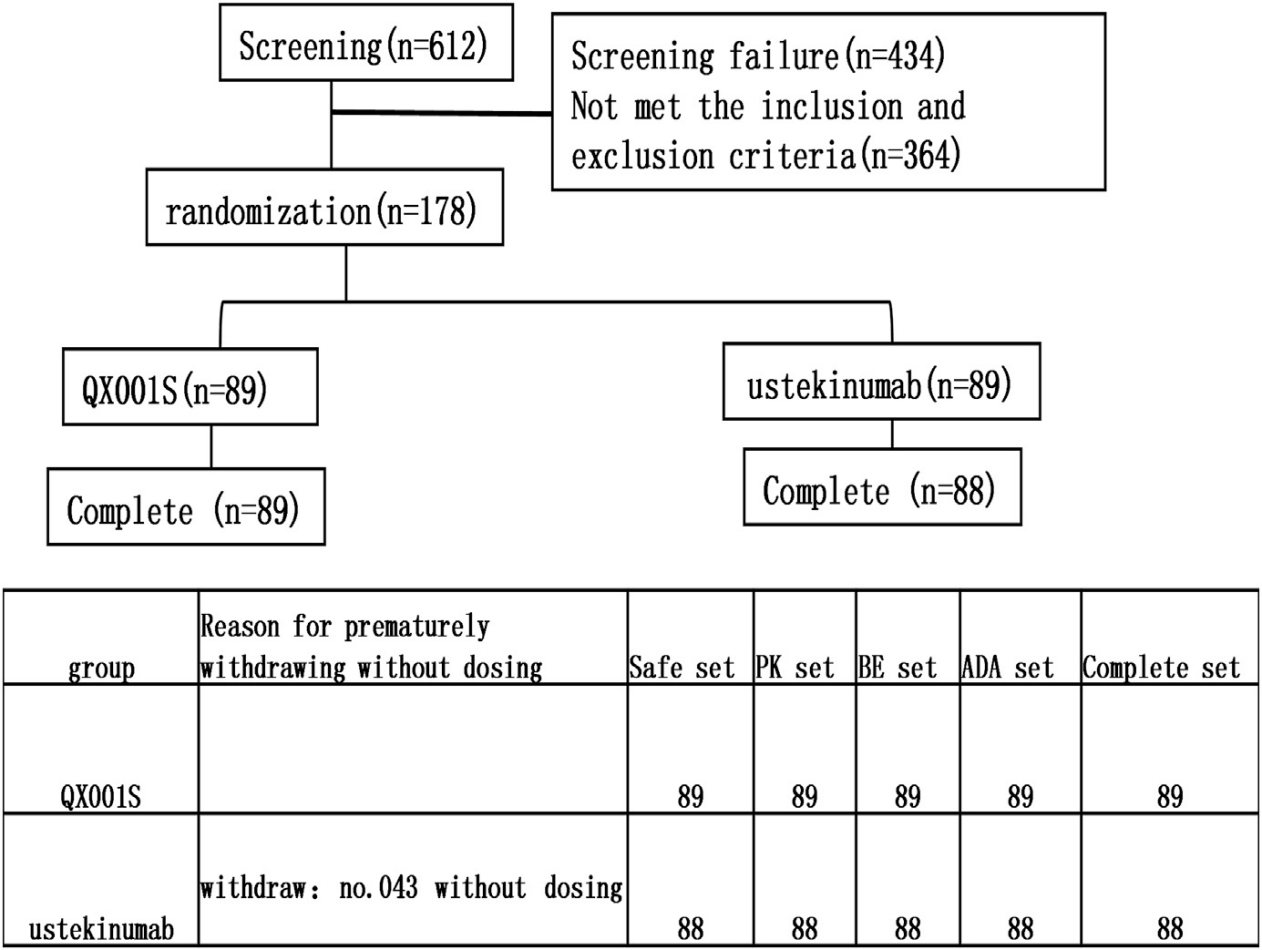
The flow chart for the study and reasons for premature withdrawal before dosing.

This study was designed as a randomized, double-blind, single-dose, two-arm, parallel clinical trial with healthy Chinese men. The purpose of the study was to evaluate the bioequivalence of the proposed biosimilar (QX001S, 45 mg) with its reference drug (ustekinumab, Starino®, a trademark in Europe). The bioequivalence study/investigation included the evaluation of the tolerance, immunogenicity, and PK of the biosimilar and reference drugs. Overall, 178 eligible participants were randomly assigned and stratified into two treatment groups based on body weight (50 to <67.5 kg and ≥67.5 to ≤85 kg) at a 1:1 ratio. The participants in each group received a single subcutaneous injection of 45 mg QX001S or ustekinumab. Individuals in each of the prespecified weight groups were equally assigned to the two treatment groups through randomization (Figure 1).

The inclusion criteria for participants were as follows: 1) healthy men aged between 18 and 50°years; 2) body mass index of 18.0–28.0 kg/m^2^; 3) total body weight between 50 and 85 kg; and 4) normal blood and urine routine as well as hepatic and renal function test results during enrollment or slight abnormality without clinical significance. Only participants (including their partners) with no fertility plans for the next 6°months who voluntarily used effective contraceptive measures were included.

The main exclusion criteria were as follows: 1) allergic constitution (a variety of drugs and food allergies) or allergic to the study drugs; 2) blood donation or substantial blood loss (>450 ml) within 3°months prior to the study, or plans for blood donation or surgery during the study period; 3) abnormal electrocardiograph (ECG) with clinical significance; 4) abnormal clinical laboratory examination results with clinical significance; 5) a history of invasive systemic fungal infection or other opportunistic infections within 2°months prior to screening; 6) systemic or local infections, such as risk of sepsis and/or known active inflammation, within 2°months prior to or during the screening showing C-reactive protein (CRP) levels above 1.5× the normal upper limit; 7) positive results for either hepatitis (including hepatitis B and C), AIDS, or syphilis; 8) use of any biological products within 3°months prior to the screening; 9) use of any immunoglobulins in the previous year; 10) receiving live (attenuated) vaccine within 12°weeks prior to the screening or during the period of this study; and 11) planning to receive any biological drugs within 3°months or monoclonal antibody drug within 9°months after administration of the study drug.

Participants were admitted to the research center the day before the injection of the test drugs. For safety assessment, each participant was asked to stay in the research center for at least 5°days after receiving the investigational product (IP). All participants were also followed up for the assessment of PK, immunogenicity, and safety for 113°days after administration. For safety reasons, the sentinel staggered dosing method was adopted in this study. This dosing method is often adopted during early phases of single ascending dose (SAD) trials and involves sequential IP administration in staggered cohorts with an appropriate period of observation between dosing individual participants. In our study, the participants were initially administered the IP in staggered cohorts, with the first and second cohorts comprising two and four participants, respectively. These participants were asked to stay at the research center for at least 9°days after the initial dose of IP for safety evaluation. Based on the sentinel safety results, the principal investigator determined whether subsequent participants would be monitored in either sentinel mode or routine follow-up mode.

All participants were administered (by subcutaneous injection) a single dose (45 mg) of QX001S or ustekinumab produced by Qyuns Therapeutic Co. Ltd (batch number: F20180101) and Starino®, Janssen-Cilag International N.V. (batch number: IASIDMS), respectively. Screening was performed 14°days before the drug dosing date. The participants were asked to fast for at least 8 h before the administration of the drugs.

### Evaluation of PK Parameters

For PK evaluation, blood samples were collected from the participants at 1 h before the administration (predose) and at 4, 12, 24 h (day 2), 48 h (day 3), 96 h (day 5), 120 h (day 6), 144 h (day 7), 168 h (day 8), 192 h (day 9), 216 h (day 10), 240 h (day 11), 288 h (day 13), 336 h (day 15), 504 h (day 22), 672 h (day 29), 1,008 h (day 43), 1,344 h (day 57), 1,680 h (day 71), 2016 h (day 85), 2,352 h (day 99), and 2,688 h (day 113) after administration.

The serum levels of ustekinumab and QX001S were determined using enzyme-linked immunosorbent assay (ELISA) provided by the Junke Zhengyuan (Beijing) Pharmaceutical Research Co., Ltd. (Beijing, China) (Supplementary material).

PK parameters were calculated using a non-compartmental analysis model, for which the concentration-time data were the time to peak (T_*max*_), maximum observable serum concentration (C_*max*_), clearance (CL), volume of distribution (Vz), half-life (*t*_1/2_), and area under the curve (AUC) from zero to the final quantifiable concentration (AUC_0–*t*_) and to infinity (AUC_0–∞_). The actual collection time for each sample was used for the analysis of PK parameters. The PK parameters were analyzed using Phoenix WinNonLin®, an internally validated software system (v8.0, Pharsight Corporation, Certara, L.P. Princeton, New Jersey, United States).

LambdaZ was the first-order rate constant associated with the terminal (log-linear) portion of the curve, estimated from the linear regression of time vs. log concentration using the best fit method. The clearance (CL) = dose/AUC0-∞; volume of distribution (Vz) = dose/(AUC0-∞* lambdaZ); and half-life = ln (2)/lambdaZ. AUC0–∞ was calculated as follows: AUC from the time of dosing extrapolated to infinity, based on the last observed concentration (Clast). AUC0-∞ = AUC0-t + Clast/lambdaZ.

#### Immunogenicity Evaluations

The time points for blood sample collection were 1 h before administration and 8, 15, 29, 57, and 113°days after administration. In this study, anti-drug antibodies (ADAs) were detected with an electrochemiluminescence immunoassay (ECLIA). It would have been necessary to further examine the presence of neutralizing antibodies (NAbs) in participants with positive ADA test results who had either antibody-related adverse reactions or significantly abnormal PK values. However, the NAb test was not performed in this study because none of the participants met these criteria.

#### Tolerance Evaluations

On days 3, 5, 9, 15, 22, 43, 85, and 113 after administration, the following examinations were performed: hematology, coagulation routine, biochemistry, and urinalysis tests. ECG screening was also conducted at different time points after administration, specifically at 4 h, 12 h (first day), and 2, 3, 5, 7, 8, 9, 10, 15, 22, 43, 85, and 113°days. To monitor adverse events (AEs), additional examinations were performed, such as vital sign, physical examination, electrocardiogram, urinalysis, and chemistry tests. The AEs were recorded and their grades were assessed according to the National Cancer Institute Common Terminology Criteria for AEs (CTCAE; V.5.0). Participants with AEs were observed until they reached normal or acceptable stability, as determined by both the main investigator and sponsor, or were lost to follow-up.

### Sample Size Estimation

The appropriate sample size for each group was estimated using the following parameters: 1) geometric mean ratio (GMR of C_max_ and AUC for the test drug vs. the reference drug), 2) power (1−β), 3) significance level (two-sided α = 5%), and 4) inter-subject variability (inter-CV) of the C_max_ and AUC. The appropriate sample size (initial: 142; inter-CV of C_*max*_ for ustekinumab: 35.8%, data from (Centocor Ortho Biotech Inc, 2020) was determined with a computer program (NQuery 8.3.0.0, Boston, United States) according to recent FDA guidelines. The GMR of the C_*max*_ and AUC for the test drug against the reference drug was set at 95% to obtain 90% power (1−β) at a significance level (two-sided α) of 5% (Julious, 2004). The inter-CV was represented by the coefficient of variation (CV). The final sample size for this study was 178 (89 participants per group) based on the above calculations and the calculated drop-out rate was 20%.

### Statistical Analysis

The variance of PK parameters, such as Cmax, AUC_0–t_, and AUC_0–∞_ was analyzed using the least squares method, but the data required logarithmic transformation before variance analysis. If the 90% confidence intervals (CI) for the ratio (test/reference) of the mean AUC and Cmax (log-transformed) fall within the range of 80–125%, a biosimilar is considered to have pharmacokinetic biosimilarity to its reference product. The statistical analysis methods used in this study were the chi-squared test for categorical variables, *t*-test for normally distributed data, and Wilcoxon rank-sum test (Mann–Whitney test) for data with an unknown distribution. SAS 9.4 software (SAS Institute Inc. Cary, NC, United States) was used for the statistical analysis of data in this study. A *p-*value of <0.05 was considered statistically significant.

## Results

### Participants

A total of 612 healthy men were screened and 178 participants were finally recruited for the study. However, only 177 received the assigned study drug because one participant in the ustekinumab group withdrew from the study due to family issues (Fig. 1). Finally, 177 participants were included in the analysis of safety, PK, BE, and immunogenicity (ADA) (Figure 1). The demographic and baseline characteristics of the participants in the two treatment groups were similar, with *p* > 0.05 (Table 1).

**TABLE 1:** Demographic and baseline characteristics.

	QX001S group	Ustekinumab group	Total	*p*
	(*n* = 89)	(*n* = 88)	(*n* = 177)	
Age (year), mean (SD)	35.7 (8.00)	35.7 (7.72)	35.7 (7.84)	0.99
Ethnicity (han, n [%])	82 (92.1%)	85 (95.5%)	167 (93.8%)	0.19
Weight (kg), mean (SD)	65.54 (6.751)	66.20 (6.921)	65.87 (6.825)	0.52
BMI (kg/m^2^), mean (SD)	23.22 (2.392)	23.36 (2.370)	23.29 (2.375)	0.69

Abbreviation: BMI, body mass index; SD, standard deviation

### PK Evaluations

**FIGURE 2:**
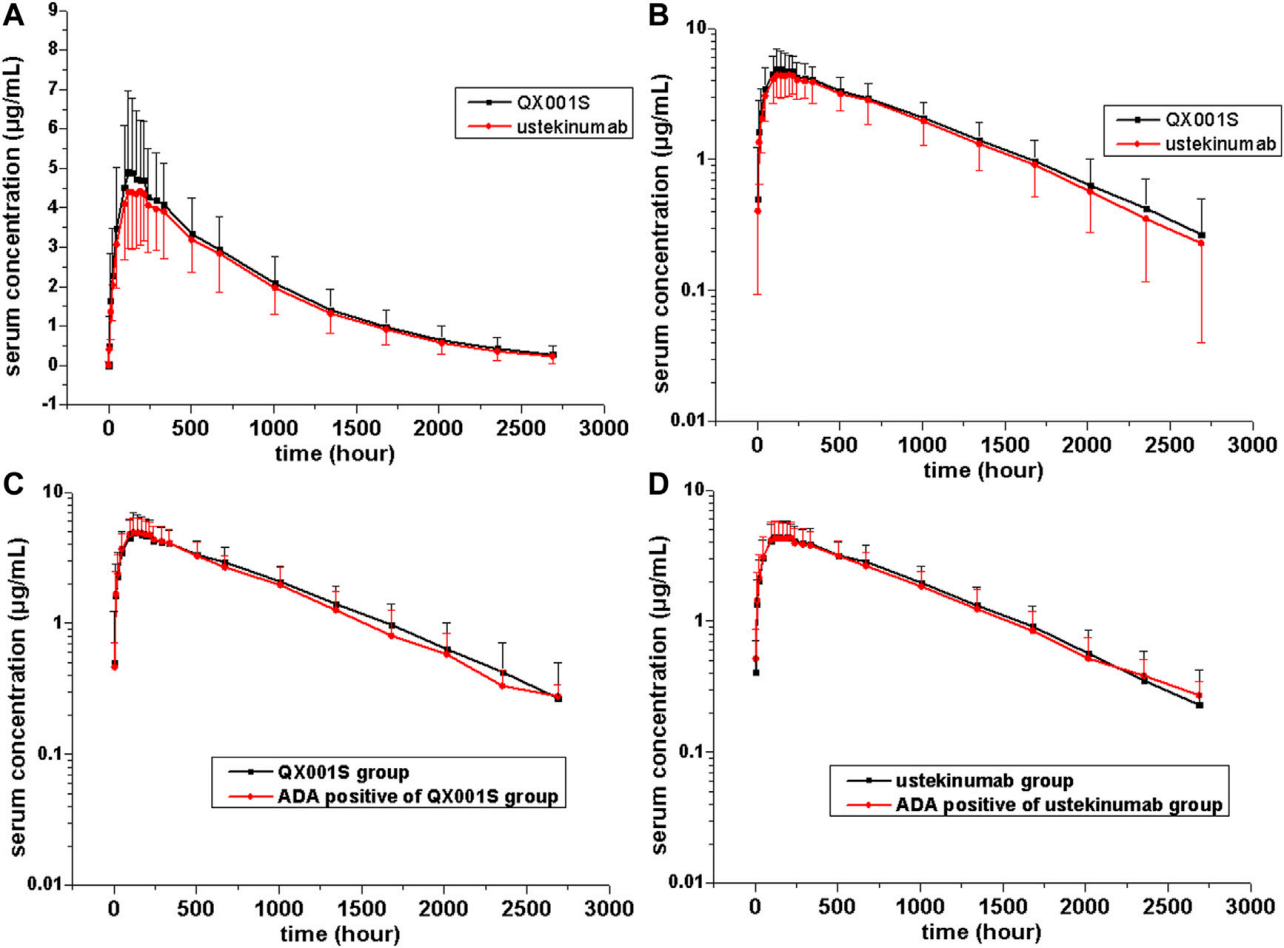
Serum drug concentration-time profiles for ustekinumab and its biosimilar. Mean ± standard deviation values **(A)**; log10 mean ± standard deviation values **(B)**; Mean ± standard deviation values of ADA-positive and total individuals in the QX001S **(C)** and ustekinumab **(D)** groups.

**FIGURE 3:**
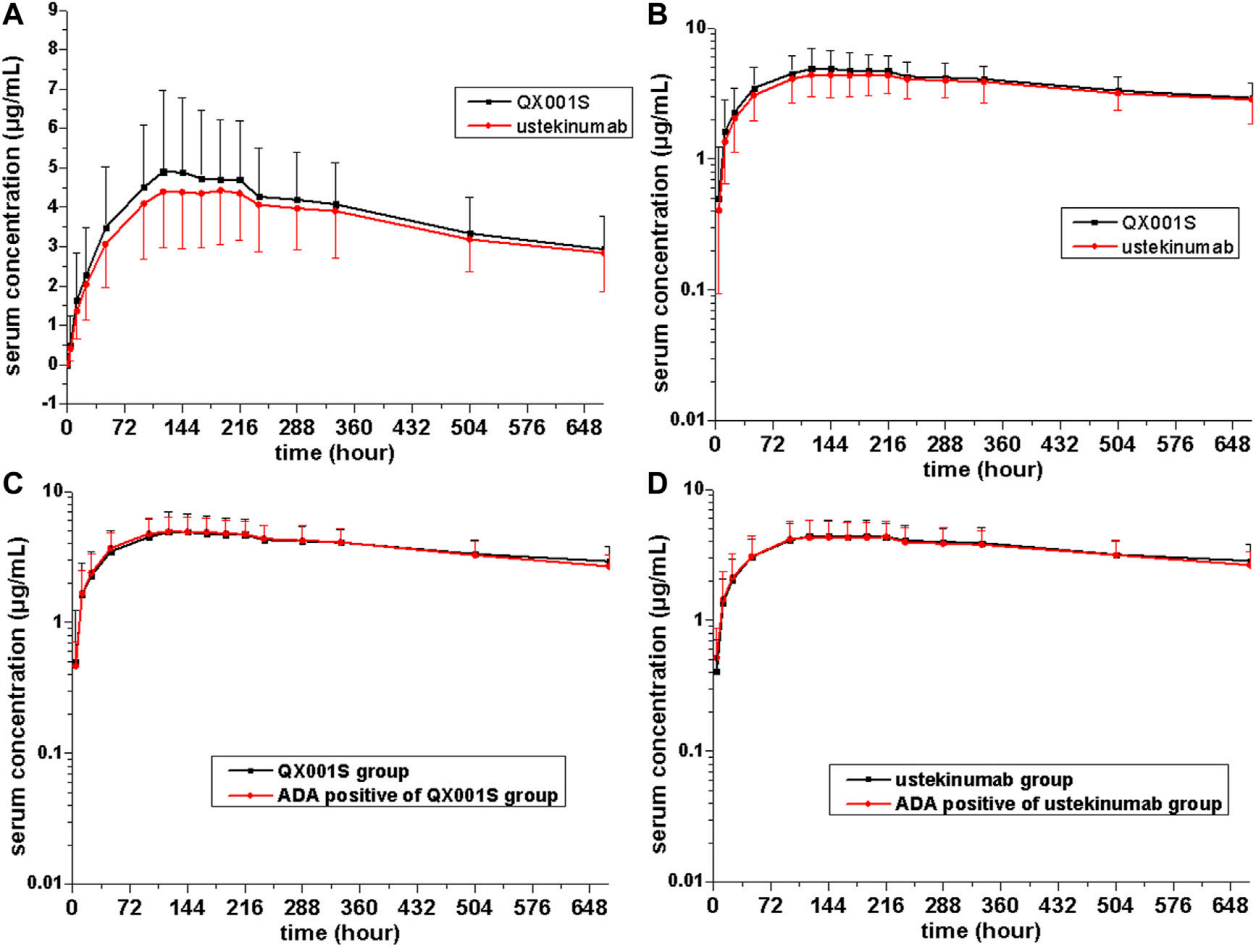
Serum drug concentration-time profiles for ustekinumab and its biosimilar for 0–672 h after dosing. Mean ± standard deviation values **(A)**; log10 mean ± standard deviation values **(B)**; Mean ± standard deviation values for ADA-positive and total individuals in the QX001S **(C)** and ustekinumab **(D)** groups.

The mean serum concentration-time curves after a single dose of ustekinumab and its biosimilar in healthy Chinese men (*n* = 177) showed a slowly increasing phase after administration, followed by a slowly declining phase at higher concentrations and a slightly faster elimination phase at lower concentrations (Figures 2, 3).

PK analysis of ustekinumab and its biosimilar showed slow clearance, long *t*_1/2_, and small Vz values in the non-compartmental analysis model. The median T_*max*_ values were similar between the two groups at 168 h after administration. The estimated geometric mean *t*_1/2_ values were comparable between the two treatment groups, ranging from 577.9 to 580.6 h. The clearance (CL) and Vz values in the two groups were also comparable. Other PK parameters such as the mean C_*max*_, AUC_0–*t*_, and AUC_0–∞_ estimates in the two groups were also similar (*p >* 0.05, Table 2, Supplementary table S1, Figures 2, 3).

**TABLE 2:** Comparison of pharmacokinetic parameters between the QX001S and ustekinumab groups (Mean ± SD or median [min, max]).

	QX001S group (*n* = 89)	Ustekinumab group (*n* = 88)	*p*	Inter-CV	GMR (90%CI)	GMR (90%CI)^a^	GMR (90%CI)^b^	Re-estimated size
T_max_ (h)^c^	168.000 (48.00, 504.00)	168.000 (96.00, 336.93)	0.49					
C_max_ (µg/ml)	5.3838 ± 1.9603	4.8836 ± 1.5234	0.06	33.5	109.43 (100.90, 118.68)	109.36 (100.67, 118.80)	106.58 (96.56, 117.65)	196
AUC_0-t_ (h^c^μg/mL)	4,816.6552 ± 1,489.9288	4,516.1668 ± 1,357.5743	0.17	32.0	106.66 (98.71, 115.26)	106.62 (98.53, 115.36)	103.45 (94.41, 113.37)	124
AUC_0-∞_ (h•μg/mL)	5,186.4298 ± 1749.6969	4,840.9347 ± 1,520.0368	0.18	33.5	106.8 (98.49, 115.81)	106.59 (98.17, 115.72)	103.45 (93.90, 113.98)	134
t_1/2_ (h)	577.9 ± 136.98	580.6 ± 155.18	0.90					
Vz (L)	7.6974 ± 2.1457	8.2152 ± 2.3119	0.12					
CL (L/h)	0.0096 ± 0.0033	0.0103 ± 0.0036	0.17					

c Median [min, max].

a QX001S/ustekinumab after excluding a participant that was ADA-positive pre-dosing.

b QX001S/ustekinumab after excluding a participant that was ADA-positive pre and post-dosing; geometric mean ratio (GMR).

As shown in Table 2, the PK parameters were comparable between the two treatment groups; in particular, the key characteristics of PK i.e. the 90% CIs of the ratios for C_*max*_, AUC_0–*t*_, and AUC_0–∞_ were 100.90–118.68%, 98.71–115.26%, and 98.49–115.81%, respectively, for QX001S vs. Ustekinumab, which were within the predefined bioequivalence limits, i.e. ranging from 80.00 to 125.00%, indicating pharmacokinetic biosimilarity between those two drugs. Since a larger inter-CV indicates a broader 90% CI, the sample size was re-estimated using the bioequivalence analysis data (GMR and inter-CV). Thus, the re-estimated value was closer to the number of recruited participants.

### Immunogenicity Evaluations

In this study, before administration of the IPs, ADA was detected in several participants in the QX001S group (two participants) and ustekinumab group (three participants). In total (before and after administration), 17 participants in the QX001S group (19.1%, 17/89) and 36 in the ustekinumab group (40.9%. 36/88) were positive for ADA. Evaluations showed that the ADA-positive rates increased over time, especially by days 57 (1,344 h) and 113 (2,688 h). It is notable that in both the QX001S and ustekinumab groups, the positive rates for the ADA test increased as the drug concentration declined. However, the ADA-positive rates were lower in the QX001S group than in the ustekinumab group (Table 3).

**TABLE 3:** Summary of immunogenicity (anti-drug antibody, ADA) assessment (number [%] of participants with positive ADA).

Time (hour (day)	QX001S group (*n* = 89)	Ustekinumab group (*n* = 88)	*p*
Pre-dose	2 (2.2)	3 (3.4)	0.64
168 (8)	1 (1.1)	15 (17)	<0.05
336 (15)	3 (3.4)	14 (15.9)	<0.05
672 (29)	3 (3.4)	11 (12.5)	<0.05
1,344 (57)	11 (12.4)	15 (17)	0.37
2,688 (113)	11 (12.4)	21 (23.8)	<0.05

The mean serum concentration-time curves for the ADA-positive and total participants were similar in the two treatment groups (Figure 2). The analysis of ADA-related sensitivity showed the results were consistent with the bioequivalence analysis data as mentioned above, supporting the conclusion regarding the pharmacokinetic biosimilarity between two treatment groups. After removing the data for ADA (+) participants before dosing (five participants) and the data for all ADA (+) participants in this study, the re-calculated 90% CIs for C_*max*_, AUC_0–*t*,_ and AUC_0–∞_ in the two groups were within the pharmacokinetic biosimilarity limits of 80.00–125.00% (Table 2).

### Safety Evaluations

In this study, there were no serious AEs (SAEs), discontinuations, or deaths due to AEs. Overall, 75 (42.4%) participants experienced adverse reactions, specifically 38 participants (42.7%) in the QX001S group and 37 (42.0%) in the ustekinumab group (Table 4). The overall incidence rates for adverse reactions in the two groups were similar (*p* > 0.05). However, in the QX001S group, an increase in the alanine aminotransferase level was found in one participant with a grade III adverse reaction (*n* = 1 [1.1%]). Other adverse reactions were classified as Grades I and II according to the common terminology criteria for AEs (CTCAE). The most common adverse reactions (>4%) were upper respiratory infection and an increase in the parameters for the clinical lab tests, such as the alanine aminotransferase level, leukocyte count, neutrophil count, aspartate aminotransferase level, and triglyceride level (Table 4).

**TABLE 4:** Adverse reactions (number of reactions, number [%] of participants, more than 4%).

	QX001S group (*n* = 89)		Ustekinumab group (*n* = 88)		Total (*n* = 177)		
	n (%)	[Number of reactions]	n (%)	[Number of reactions]	n (%)	[Number of reactions]	*p*
Total	38 (42.7)	88	37 (42.0)	55	75 (42.4)	143	0.93
Upper respiratory infection	9 (10.1)	9	6 (6.8)	6	15 (8.5)	15	0.43
Elevated triglyceride level	3 (3.4)	4	6 (6.8)	6	9 (5.1)	10	0.29
Elevated leukocyte count	5 (5.6)	6	3 (3.4)	3	8 (4.5)	9	0.47
Elevated alanine aminotransferase	12 (13.5)	14	4 (4.5)	4	16 (9.0)	18	0.04
Elevated aspartate aminotransferase	8 (9.0)	9	0 (0.0)	0	8 (4.5)	9	NA
Elevated neutrophil counts	5 (5.6)	6	3 (3.4)	3	8 (4.5)	9	0.47

One participant in the ustekinumab group experienced injection site swelling after administration on day 1, but the swelling disappeared by the next day without treatment. The rest of the participants had no injection site reactions. To treat the AEs observed, 15 participants (seven in the QX001S group and eight in the ustekinumab group) transiently (one or two times) received medications such as acetaminophen tablets, cefixime, roxithromycin, and oseltamivir phosphate capsules, mostly for the control of upper respiratory infection. Taken together, no relationship between ADA development and adverse reactions was found in this study. All the adverse reactions in this study were reported to the Institutional Review Board of The First Hospital of Jilin University.

## Discussion

The pharmacokinetic biosimilarity of QX001S with its reference ustekinumab (Starino®), when administered (single subcutaneous injection) at a dose of 45 mg, was confirmed in this single-dose, randomized, phase I study. The comparison of PK parameters (Cmax and AUC) between QX001S and ustekinumab recipients using ANOVA showed that the 90% CI of GMR for the log-transformed PK parameters were within the range of 98.49–118.68%, matching the predetermined pharmacokinetic biosimilarity range of 80–125%. Other PK parameters such as T_*max*_ and *t*_½_ were also similar between the QX001S and ustekinumab groups. The safety profiles of the two treatment groups were very similar (no SAEs in this study and most AEs were mild to moderate in severity), which means both IPs (QX001S and ustekinumab) in this study were well tolerated by healthy Chinese men. Our findings pave the way for further QX001S clinical trials in the next phase according to the guidelines issued by the government and international organizations (European Medicines Agency, 2014; US Food and drug Administration, 2015; World Health Organization, 2015; Yoshihara, et al., 2021).

There are several factors that can distinguish the characteristics of biosimilars from those of small molecule compounds. These factors include the limited vascular permeability of biospecific antibodies, the recycling of new Fc receptors, and more frequent elimination of the internalized complex mediated by receptors (Schropp, et al., 2019). Our data revealed that the mean serum concentration-time curves after injection of a single dose of ustekinumab and its biosimilar demonstrated a slowly increasing phase after administration, followed by a slowly declining phase at higher concentrations, and then a slightly faster elimination phase at lower concentrations. The boundary interval for the last two phases was approximately 672 h after administration (Figure 2). The PK behavior of both QX001S and ustekinumab also follows a similar trend, i.e. when the drug concentrations decreased to relatively low levels, the last-step elimination was enhanced, perhaps due to desaturated receptor binding (Schropp, et al., 2019). The clinical trial dose, analysis of the active substance, pharmacokinetic data, safety testing, and tolerance in this study and in the study by Zhu and Weber were exactly the same (Weber and Keam, 2009; Zhu, et al., 2013).

In our study, the median T_*max*_ values were 168 h (8°days) after the administration (single dose of 45 mg) of ustekinumab and its biosimilar. However, in another study involving individuals with psoriasis, the median time to reach the maximum serum concentration (T_*max*_) was 13.5°days after a single subcutaneous administration of 45 mg of ustekinumab, which was relatively longer than ours (Centocor Ortho Biotech Inc, 2020). The T_*max*_ values reported in the previous study reflects the slow absorption characteristics of ustekinumab. The estimated geometric mean *t*_1/2_ values for both ustekinumab and QX001S were comparable, ranging from 577.9 to 580.6 h (23–24°days). These values were also shorter than the *t*_1/2_ (45.6 ± 80.2°days) in a previous psoriasis study (Centocor Ortho Biotech Inc, 2020).

In the literature, body weight was considered as an important covariate that affects the PK characteristics of ustekinumab. For individuals with a body weight >100 kg, the median concentration of ustekinumab was lower than that in individuals with a body weight ≤100 kg after treatment at same drug dose (Centocor Ortho Biotech Inc, 2020). Based on this pattern, in this study the weight stratification (50 to <67.5 kg and ≥67.5 to ≤85 kg) was adopted to minimize the changes in the PK parameters of the participants, although all of them weighed <100 kg. It was also found that in this study the subjects with higher body weight (≥67.5 to ≤85 kg) had larger volume of distribution and higher drug clearance, indicating existence of rapid drug metabolism and relatively shorter t1/2 in those subjects, which ultimately led to the decrease of exposure level, such as Cmax and AUC, as compared to those with lower weight. On the whole, Tmax is similar in both high and low weight groups, indicating that the absorption phase is similar in both of them. There were no statistically significant differences of Tmax and t1/2 between the high and low weight groups, while the other PK parameters had statistically significant differences between two drug groups (Supplementary table S2). Furthermore, the inter-CV for ustekinumab in this study was relatively high (>30%). Therefore, a larger sample size of 124–196 individuals or 62–98 participants per arm should be considered for future studies (Julious, 2004).

Since the targets of ustekinumab are IL-12 and IL-23, which are naturally occurring cytokines involved in inflammatory and immune responses, such as natural killer cell activation and CD4^+^ T-cell differentiation and activation, the main function of ustekinumab is to inhibit IL-12 and IL-23-mediated signaling pathways. However, the use of ustekinumab may increase the risk of infections and the reactivation of latent infections, malignancies, *tuberculosis*, etc. The most commonly reported adverse reactions to ustekinumab are nasopharyngitis, headache, upper respiratory tract infection, and fatigue (Centocor Ortho Biotech Inc, 2020) (Hanauer, et al., 2020). It has been reported that 61% of patients treated with ustekinumab had infections in the controlled and non-controlled portions of psoriasis clinical trials (Centocor Ortho Biotech Inc, 2020), consistent with the high incidence of infection-related adverse reactions found in our study, such as upper respiratory infection and increased leukocyte and neutrophil counts.

It is notable that the levels of alanine aminotransferase and aspartate aminotransferase were elevated with high incidence in this study, especially in the QX001S group. However, the CTCAE grades were not high and most AEs were grades I or II, with the exception of one participant with elevated alanine aminotransferase and aspartate aminotransferase (No. 090 in QX001S group). The original level of alanine aminotransferase was 12.6 U/L, but increased to 173 U/L on day 5, reached the peak value (278 U/L) on day 9, decreased to 56 U/L after treatment with silybin capsule for 8°days, and finally returned to a normal level on day 35. The majority of adverse reactions in this study did not require treatment because they automatically recovered.

The positive rate for ADA is highly dependent on the sensitivity and specificity of the detecting assay. Additionally, the positive rate may also be influenced by several factors, such as sample handling, timing of sample collection, concomitant medications, and underlying disease (Centocor Ortho Biotech Inc, 2020). The ADA-positive rate for ustekinumab in previous reports was 5% (38/743), the negative rate was 47% (351/743), and the inconclusive rate was 48% (354/743) in a study on plaque psoriasis patients treated with ustekinumab for 28–48°weeks, with the final ADA test at week 52 after administration (Armstrong and Read, 2020; Centocor Ortho Biotech Inc, 2020). In this study, the maximum ADA positive rates estimated at 113°days (16°weeks) after administration were 12.4% (QX001S) and 23.8% (ustekinumab), in agreement with those (ADA positive rate plus the inconclusive rate) reported in the above mentioned study.

It should be noted that the ADA positive rate for QX001S in this study was much lower than that for ustekinumab, implying that the occurrence of ADA does not affect the drug concentrations and bioequivalence results (Figure 2; Table 2). In this study, no acute or delayed anaphylaxis was found in the ADA-positive participants, suggesting the lack of drug-specific immunogenicity. Although the effects of drug-related immunogenicity on the safety and PK characteristics of the drug were not confirmed in this study, the immunogenicity and efficacy of both QX001S and ustekinumab should be carefully investigated in further phase III studies. Several factors should be considered in these future studies such as a larger population, multiple doses, and longer study duration (Armstrong and Read, 2020; Zhang, et al., 2020). Based on our observation of the safety and tolerance of the two drugs, the severity of all AEs in this study was mild to moderate and no SAE was reported, indicating that both QX001 S and ustekinumab are well tolerated (Hanauer, et al., 2020; Rowan, et al., 2020).

## Conclusion

This study ratified the pharmacokinetic biosimilarity between QX001S and its reference ustekinumab (Starino®). Additionally, the inter-CV for ustekinumab was high among the participants in this study. These findings support the clinical development of QX001S as a biosimilar for ustekinumab.

## Data Availability

The original contributions presented in the study are included in the article/Supplementary Material, further inquiries can be directed to the corresponding author.
