# Supplementary material for: A Biosimilarity Study Between QX001S and Ustekinumab in Healthy Chinese Male Subjects
Source: Front Pharmacol. 2021 May 18;12:675358. doi: 10.3389/fphar.2021.675358 (PMC8167066; doi:10.3389/fphar.2021.675358)
Supplement: Supplementary file 1 [file Table1.doc]

**Supplement**

**Pharmacokinetic and immunogenicity evaluations**

The enzyme-linked immunosorbent assay (ELISA) and electrochemiluminescence immunoassay analyzer (ECLIA) methods were used to measure the serum concentrations of Ustekinumab, anti-drug antibody (ADA), and [neutralizing](javascript:void(0);) [antibody](javascript:void(0);) (NAb) at the Junke Zhengyuan (Beijing) Pharmaceutical Research Co., Ltd (Beijing, China). First, venous blood samples (4 ml per sample) were collected at each time point to measure the serum Ustekinumab concentration and detect the ADA, respectively. After collection, the blood samples were allowed to clot for 30 min at room temperature and centrifuged at 1500–2000 g for approximately 15 min at 2–8°C. The serum was stored at −70°C for further analysis. The concentration range for analyzing the PK parameters was 150-5000 ng/mL, and the lower limit of quantification (LLOQ) was 150 ng/mL. For PK analysis, concentrations less than the LLOQ were set to zero. The accuracy of the inter-run assay should be within the range of 0% to 2.2%, which was expressed as the percentage relative error for the quality control samples. In the ADA test, the accuracies of the negative control, low concentration positive quality control (50 ng/mL), and high concentration quality control (2000 ng/mL) were 10.8%, 5.4%, and 12.4%, respectively. All met the acceptable standard (<15.8%).

Supplement table 1. Comparison of pharmacokinetic parameters between the groups of QX001S and Ustekinumab

|  | **QX001S group (n = 89)** | | **Ustekinumab group (n = 88)** | |  |
| --- | --- | --- | --- | --- | --- |
|  | Mean ± SD or median [min, max] | **90% CI** Mean or median | Mean ± SD or median [min, max] | **90% CI** Mean or median |  |
| **T_max_ (h)*** | **168.000 (48.000, 504.000)** | **144.000, 168.000** | **168.000 (96.000, 336.930)** | **156.000, 192.000** |  |
| **C_max_ (µg/mL)** | **5.3838±1.9603** | **5.0384, 5.7293** | **4.8836±1.5234** | **4.6136, 5.1536** |  |
| **AUC_0-t_ (h*μg/mL)** | **4816.6552±1489.9288** | **4554.1161, 5079.1943** | **4516.1668±1357.5743** | **4275.565, 4756.7686** |  |
| **AUC_0-∞_ (h•μg/mL)** | **5186.4298±1749.6969** | **4878.1171, 5494.7424** | **4840.9347±1520.0368** | **4571.5398, 5110.3296** |  |
| **t_1/2_ (h)** | **577.9±136.98** | **553.8, 602.1** | **580.6±155.18** | **553, 608.1** |  |
| **Vz (L)** | **7.6974±2.1457** | **7.3194, 8.0755** | **8.2152±2.3119** | **7.8055, 8.6249** |  |
| **CL (L/h)** | **0.0096±0.0033** | **0.0091, 0.0102** | **0.0103±0.0036** | **0.0097, 0.011** |  |

*Median [min, max];

Supplement table 2. Comparison of pharmacokinetic parameters between the groups of 50 to < 67.5 kg and≥ 67.5 to ≤ 85 kg

|  | 50 to < 67.5 kg | | ≥ 67.5 to ≤ 85 kg | | p |
| --- | --- | --- | --- | --- | --- |
|  | Mean ± SD or  median [min, max] | 90% CI Mean  or median | Mean ± SD or  median [min, max] | 90% CI Mean  or median |  |
| QX001S group |  |  |  |  |  |
| n | 47 |  | 42 |  |  |
| T_max_ (h)* | 144(96, 336.87) | 144.000, 168.000 | 168(48, 504) | 144.000, 192.000 | 0.3625 |
| C_max_ (µg/mL) | 6.2649±2.095 | 5.7519, 6.7779 | 4.3979±1.1994 | 4.0864, 4.7093 | <.0001 |
| AUC_0-t_ (h*μg/mL) | 5466.3212±1563.2866 | 5083.5383, 5849.1041 | 4089.648±998.7381 | 3830.302, 4348.994 | <.0001 |
| AUC_0-∞_ (h•μg/mL) | 5943.0136±1893.3962 | 5479.4008, 6406.6265 | 4339.7764±1076.2262 | 4060.3088, 4619.2441 | <.0001 |
| t_1/2_ (h) | 592.7±160.88 | 553.3, 632.1 | 561.4±103.4 | 534.5, 588.2 | 0.2736 |
| Vz (L) | 6.7629±1.7532 | 6.3336, 7.1922 | 8.7432±2.0765 | 8.204, 9.2824 | <.0001 |
| CL (L/h) | 0.0084±0.0027 | 0.0077, 0.009 | 0.0111±0.0033 | 0.0102, 0.0119 | <.0001 |
| Ustekinumab group |  |  |  |  |  |
| n | 47 |  | 41 |  |  |
| T_max_ (h)* | 168(96, 336.7) | 144.000, 191.759 | 191.5(96, 336.93) | 144.000, 192.000 | 0.7713 |
| C_max_ (µg/mL) | 5.6368±1.4958 | 5.2705, 6.0031 | 4.0202±1.0216 | 3.7516, 4.2889 | <.0001 |
| AUC_0-t_ (h*μg/mL) | 5117.0485±1369.8004 | 4781.6422, 5452.4548 | 3827.3512±969.6203 | 3572.3669, 4082.3355 | <.0001 |
| AUC_0-∞_ (h•μg/mL) | 5478.4403±1553.6824 | 5098.0091, 5858.8715 | 4110.1355±1106.5216 | 3819.1498, 4401.1212 | <.0001 |
| t_1/2_ (h) | 582.6±178.39 | 539.0, 626.3 | 578.1±125.55 | 545.0, 611.1 | 0.7035 |
| Vz (L) | 7.0495±1.2671 | 6.7393, 7.3598 | 9.5515±2.5202 | 8.8887, 10.2142 | <.0001 |
| CL (L/h) | 0.009±0.003 | 0.0082, 0.0097 | 0.0119±0.0037 | 0.0109, 0.0128 | <.0001 |

*Median [min, max];
